# Supplementary material for: Membrane Vesicles Can Contribute to Cellulose Degradation by Teredinibacter turnerae, a Cultivable Intracellular Endosymbiont of Shipworms
Source: Microb Biotechnol. 2024 Dec 11;17(12):e70064. doi: 10.1111/1751-7915.70064 (PMC11632262; doi:10.1111/1751-7915.70064)
Supplement: Supplementary file 1 — Figure S1. [file MBT2-17-e70064-s003.docx]

# Supplemental Figures


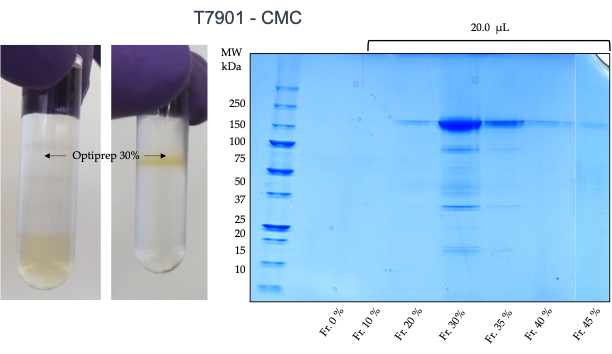


**B)**

**A)**

**After**

**Before**

Figure S1. Purification of T. turnerae crude MVs by density gradient ultracentrifugation. (A) Representative image of density gradient banding before centrifugation and sample visualization at the interface of 30% fraction. (B) Representative SDS-PAGE analysis of OMVs collected from each density fraction.
