# Supplementary material for: Membrane Vesicles Can Contribute to Cellulose Degradation by Teredinibacter turnerae, a Cultivable Intracellular Endosymbiont of Shipworms
Source: Microb Biotechnol. 2024 Dec 11;17(12):e70064. doi: 10.1111/1751-7915.70064 (PMC11632262; doi:10.1111/1751-7915.70064)
Supplement: Supplementary file 2 — Figure S2. [file MBT2-17-e70064-s001.docx]

# Supplemental Figures

kDa

**T7901**

**SR01903**


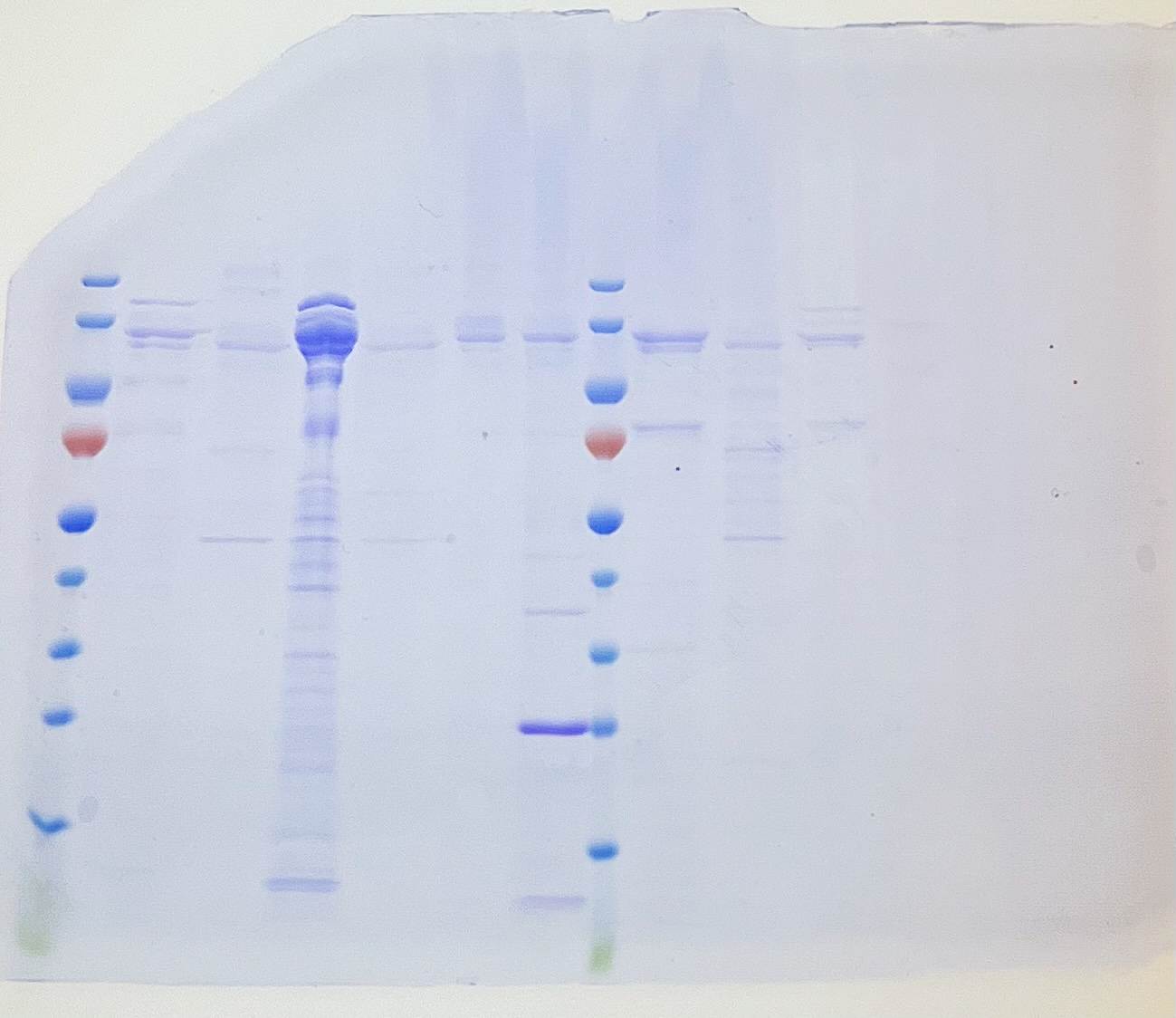

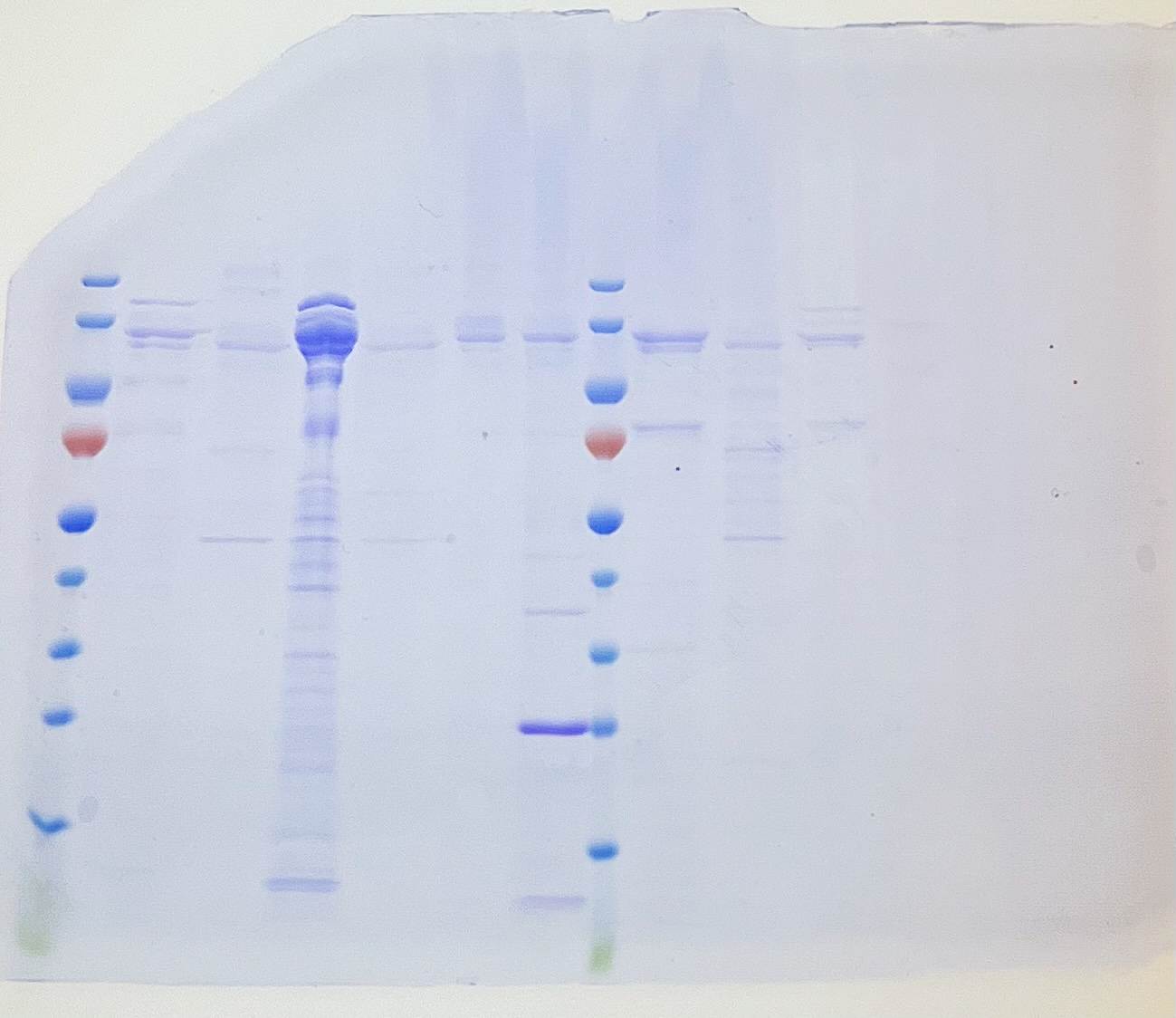


180

130

100

70

55

Figure S2. Standard SDS-PAGE gel analysis performed under denaturing and reducing conditions of MV suspensions from T. turnerae (strains T7901 and SR01903). MV suspensions were normalized by protein content as determined by Pierce 660 assay (Thermo Scientific), and 4 μg of protein was loaded in each sample lane.

40

10

15

25

35
