# Supplementary material for: Membrane Vesicles Can Contribute to Cellulose Degradation by Teredinibacter turnerae, a Cultivable Intracellular Endosymbiont of Shipworms
Source: Microb Biotechnol. 2024 Dec 11;17(12):e70064. doi: 10.1111/1751-7915.70064 (PMC11632262; doi:10.1111/1751-7915.70064)
Supplement: Supplementary file 3 — Table S1. [file MBT2-17-e70064-s002.docx]

# Supplemental Tables

Table 1. Selected details of statistical analysis performed on measured enzyme activity via DNS assay

1. Data summary from DNS assay measurements (Units/mg) used in statistical testing.

1. One-way analysis of variance (ANOVA) and Tukey multiple comparison test results that are presented in Figure 4 C.
